# Collectanea Medica, Consisting of Anecdotes, Facts, Extracts, Illustrations, Queries, Suggestions, &c.

**Published:** 1815-01

**Authors:** 


### COLLECTANEA MEDICA,

CONSISTING OF

### ANECDOTES, FACTS, EXTRACTS, ILLUSTRATIONS, QUERIES, SUGGESTIONS, &c.

RELATING TO THE

*History or the Art of Medicine, and the Auxiliary Sciences.*

---

### *On some Unknown Combinations of Chromic Acid with different Bases.** By Professor John.

I Undertook these experiments with a view of filling up some gaps which still exist in the table of chromates; but I must acknowledge that they do not possess the accuracy which might have been obtained by performing the experiments upon a larger scale. When we attempt to chrystallize salts in small quantities, we have often to struggle with insurmountable difficulties; and, even in the most favourable circumstances, it is very difficult to determine the state of small, and often imperfectly formed, crystals.

1. *Chromate of Soda.*—Chromic acid, neutralized with soda, forms a dark yellow solution, which, by spontaneous evaporation, forms thin six-sided tables, with two long and four short faces. They are transparent, easily soluble in water, and do not alter vegetable blue colours. They are not entirely insoluble in alcohol, but require a considerable proportion of that liquid to dissolve them.

2. *Chromate of Potash.*—Chromic acid dissolves potash, and at the same time lets fall a small quantity of green oxide.† The orange-coloured solution has a great tendency to efflorescence, especially when little pieces of wood are introduced into it. Chromate of potash, when rapidly evaporated, forms a yellow saline mass; when more slowly evaporated, it forms four-sided tables; they are not altered by exposure to the air, are transparent, and have an Aurora-red colour.

3. *Chromate of Ammonia.*—This compound effloresces, and forms dendritical crystals, while a brown powder separates, which is oxide of chromium. It is formed in consequence of the decomposition of the chromic acid by the ammonia, and appears as often as the salt is dissolved in water, and allowed to effloresce. When this salt is exposed to a red heat, it is completely decomposed.

---

* Translated from Schweigger's Neues Journal für Chemie und Physik, iii. 378.

† This oxide was without doubt dissolved in the chromic acid, and owed its formation to some accident, probably the filtration of the acid through paper. When this green oxide is separated by the filter, no new precipitate appears. Ammonia, on the contrary, precipitates the chromic acid.

4. *Chromate of Glucina.*—Glucina, thrown down by carbonate of potash, was dissolved very slowly by concentrated chromic acid: the solution has a yellow colour, and does not seem capable of crystallizing.

5. *Chromo-Sulphate of Glucina.*—When sulphate of glucina is poured into chromic acid, the appearance of the solution is not altered; but when the liquid is evaporated there remains behind a triple salt in dendritical crystals, in a state of efflorescence.

6. *Chromate of Yttria.*—Chromic acid dissolves yttria, cold, in considerable quantity and with effervescence. The solution has an astringent and pungent taste, and, like most of the chromates, has an orange-red colour passing into yellow. The solution is quite neutral. Chromate of yttria, gives by evaporation dendrites, which are composed of very fine and firm crystals, which, if I may be allowed a comparison, are similar to a tree loaded with fruit. These crystals, which consist of prisms and cubes, have a peculiar appearance. Chromate of yttria is very soluble in water.

7. *Chromate of Strontian.*—This salt is insoluble. When carbonate of strontian is thrown into chromic acid, a yellowish powder is formed, which is this salt: probably it would be most easily procured by double decomposition.

8. *Chromate of Barytes.*—Carbonate of barytes is not dissolved by chromic acid; but the earth combines with the acid, and forms a light yellow insoluble powder, which lies at the bottom of the vessel. This salt is best obtained by mixing together muriate of barytes and chromic acid.

9. *Chromate of Nickel.*—Chromic acid dissolves the carbonate of nickel in considerable quantity; but, after some hours, a pulverulent precipitate falls from the clear solution, which is again soluble in an excess of acid. This precipitate is probably a chromate of nickel. The acid solution gives, by slow evaporation, fern-leaved crystals in the state of elliptical plates, truncated on both sides, which towards the beginning and end of the leaf stalks become smaller, in order to preserve the resemblance. When these crystals are exposed to a high temperature, the acid is decomposed, and a black mass formed, which is insoluble in water, and consists of oxide of chromium and oxide of nickel.

10. *Chromate of Cerium.*—Carbonate of cerium is dissolved in great abundance by chromic acid. The solution has a yellow colour, and an astringent taste: after some time the chromate of cerium falls to the bottom as a yellow powder. The remaining solution deposits some small reddish transparent crystals, which make their appearance in the midst of an uncrystallizable mass.

11. *Chromate of Tellurium.*—Tellurium likewise is dissolved by the acid: of all the chromates, this salt appears least disposed to crystallize. It deposits some soft round grains, but the greatest part goes into the state of a syrupy mass.

12. *Chromate of Uranium.*—Carbonate of uranium is readily dissolved with effervescence by chromic acid. The solution is coloured yellow, has an astringent taste, and the salt is easily dissolved solved in water. It shoots into a dendritical mass containing small cubic crystals. They have an aurora colour. This salt melts in a weak red heat, and after cooling appears of a dark brown colour. This compound does not appear to be so easily decomposed as chromate of nickel; for the brown mass dissolves in water, except a small residue, which is a mixture of oxide of chromium and oxide of uranium. The solution has a yellow colour, and potash precipitates from it yellow oxide of uranium.—*Annals of Philosophy.*

---

*Remarks on Mr. Hume's Paper on Barytes, contained in the Philosophical Magazine*, vol. xiv. 1802. By Mr. R. Phillips.

Having had occasion to consult various authorities respecting the chemical properties of barytes, I was much surprised on reading the contents of a paper on the subject by Mr. Hume, printed in the Philosophical Magazine for 1802, but which I do not remember to have seen until very lately.

In this memoir some facts are stated, which the author says he "had never yet seen pointed out by any chemical writers;" and others are mentioned, which he asserts had never been previously "enumerated by any author."

The statements which I shall first notice are, "that nitrate of barytes, not in crystals only, but even a saturated aqueous solution, is perfectly insoluble in nitrous acid of the usual specific gravity;" and "that muriate of barytes is virtually insoluble in muriatic acid."

Now that these facts were well known, and had been described previously to the publication of Mr. Hume's paper, will appear by the following quotation from a memoir by Sage, contained in vol. lxxxviii. p. 145, of the Mémoires de l'Académie des Sciences for 1788:—"L'acide nitreux à trente deux degrés, fait d'abord une vive effervescence avec le spath pesant aéré : le nitre qui en resulte demandant beaucoup d'eau pour sa dissolution, se précipite aussitôt qu'il se forme, comme l'a observé M. Klaproth."

"L'acide marin dissout avec effervescence le spath pesant aéré : le sel qui en résulte se précipite aussitôt. L'acide marin qui surnage, ne tient point en dissolution le sel à base de terne pesante."

The next discovery claimed by Mr. Hume to which I shall advert is, that "sulphate of strontian has a capacity for super-saturation, forming an acidulous sulphate in solution, and decomposable by water;" and of this fact he asserts that "no author has given any account."

That this statement as to sulphate of strontian is correct, and the assertion respecting it erroneous, is proved by the following extract from p. 334, vol. i. of the English Translation of Klaproth's Analytical Essays, published in 1801; and the original was printed in Crell's Annals for 1795:—"Upon 60 grains of pulverized strontianite, introduced into a retort, I poured by degrees two ounces of concentrated sulphuric acid. The first portion that effused caused a great frothing. The contents of the retort were then brought to boiling in a sand heat. After cooling, the earth was found entirely dissolved, and the solution colourless; but it is again decomposed as soon as any water is added."

The remaining parts of Mr. Hume's paper which I shall notice are contained in the following quotations:—"Sulphate of barytes is completely soluble in sulphuric acid, forming a saline fluid or acidulous sulphate, analogous, in some of its characters, to phosphate of lime and many other salts, with capacity for excess of acid; decomposable by water alone, which returns it to simple sulphate; and this salt," says Mr. Hume, "never has been enumerated by any author."

"Carbonate of barytes is also totally decomposed by, and soluble in, sulphuric acid, forming, of course, the same acidulous sulphate. Respecting any figure this new salt may put on, I have not yet been able fully to determine; but I strongly suspect it may, under particular circumstances, be made to crystallize."

Notwithstanding the unqualified assertion by which some of these facts, as well as those respecting strontian, are accompanied, it will appear that all of them were well known, and had been repeatedly described, before the appearance of Mr. Hume's paper, in French, German, and English.

1. The solution of sulphate of barytes in sulphuric acid, the crystallization of the solution, and its decomposition by water, are all stated in a letter from Morveau to Bergman: Journal de Physique, vol. xviii. p. 299, 1781.

2. These facts, with the additional one of the decomposition of the carbonate of barytes by sulphuric acid, are all described by Dr. Withering: Phil. Trans. 1784

3. Excepting the crystallization of the solution of the sulphate of barytes, the same circumstances are related by Sage, in the paper already quoted: Memoires de l'Academie, &c. 1788.

4. With the above exception, they are also detailed in the Journal de Physique, 1788; Sage's memoir having been also printed in that work, with some alterations.

5. These facts are all mentioned by Klaproth, in Crell's Annals, 1795.

6. The solubility of sulphate of barytes in sulphuric acid, and the decomposition of the solution by water are stated by Kirwan: Mineralogy, vol. i. p. 136, 1794.

7. The experiments alluded to are all described with such perfect clearness by Klaproth, that I shall quote the passage from the English translation, vol. i. p. 234, printed in London in 1801, the year before that in which Mr. Hume's paper was published.

After describing the solution of strontian in sulphuric acid, as already quoted, he adds, "In like manner, 60 grains of witherite were combined with two ounces of strong sulphuric acid. A great effervescence ensued; and, with the assistance of boiling heat, a complete solution, as clear as water, was likewise in this case produced. Some days after, the greatest part of this solution formed formed a crystalline mass of very tender fibres. This solution was also immediately decomposed by the admixture of water, and sulphate of barytes precipitated."

The pages of the Annals of Philosophy, the Philosophical Magazine, and the Medical and Physical Journal, exhibit ample evidence of Mr. Hume's proneness to complain without having been injured, and to attack under semblance of defence: in future, he will probably be more cautious; and, considering his situation with respect to the authors I have quoted, it will evince his prudence to refrain from charging those who may hereafter even unfairly question the priority of his remaining or future discoveries, with the conduct unmeritedly ascribed to two most respectable physicians of "unjustly throwing the veil over his efforts, in order to display their own.*"—*Annals of Philosophy.*

---

### *Further Experiments and Observations on Iodine;* by Sir H. Davy, LL.D. F.R.S. V.P.R.I.

*On the triple Compounds containing Iodine and Oxygen.*—I described in my last paper the action of iodine on fixed alkaline lixivia, and the deflagrating salts it forms. In the first experiment which I made on these compounds, I employed the first crystals which fall down from moderately strong solutions of potassa and soda saturated with iodine, which had been purified by being repeatedly acted upon by distilled water: I now find that this process is not sufficient to free the triple compound from the double compound; and that to obtain them in a state of absolute purity, it is necessary to boil them repeatedly in small quantities of alcohol of specific gravity of from 8·6 to 9·2, which dissolves the double compound, but has little power of action on the triple compound.

The triple compounds, when purified, present some curious chemical phenomena, which a minute quantity of the double compound adhering to the crystals that I operated upon, prevented me from observing in the experiments I have already communicated to the Society. I shall describe these phenomena as they are produced by the triple compound of potassium, as this substance is most easily procured in considerable quantities, but, as far as I have been able to observe, the phenomena presented by the compound of sodium are precisely analogous.

The triple compound of potassium purified by alcohol is almost tasteless, has no action on vegetable colours, is very little soluble in cold water, but more soluble in hot water; when it is thrown into concentrated nitric, or sulphuric, or phosphoric acids, it has no violent action on them. By heat it may be dissolved in them, and the solutions, when saturated, congeal and form crystalline substances intensely acid. When the substance formed by the triple compound and the nitric acid is strongly heated, the nitric acid flies off, and at the temperature at which it is entirely ex-

---

* Vide Med. and Phys. Journal, vol. xxvi. p. 109.

pelled, pelled, the substance itself begins to decompose and affords a little iodine and much oxygen.

If the solution of the triple compounds in sulphuric or phosphoric acids be heated strongly at the temperature at which the acids sublime, the triple compound itself is decomposed, and it affords oxygen and iodine, and leaves acid sulphate and phosphate of potassa. If when the mixture is rendered fluid by heat, a little sugar or other combustible matter is added, there is a violent action, and iodine is disengaged with great rapidity.

The triple compound dissolves without decomposition in solution of phosphorous acid; but, on heating the solution, oxygen is attracted by it, iodine appears, and phosphate of potassa is formed.

When the triple compound is thrown into concentrated muriatic acid, there is an effervescence, the smell of chlorine is perceived, the fluid becomes yellow, and when evaporated yields the chlorionic acid.

When the solution of the hydroionic acid in water is poured upon the triple salt, iodine is instantly produced in great quantities.

Acetic and oxalic acids dissolve the triple compound without decomposing it. On heating the solution in oxalic acid, the acid becomes brown from the deposition of charcoal, and iodine immediately appears.

When the triple compound is thrown into solution of sulphurous acid, iodine is instantly produced, and sulphuric acid formed, and, if the sulphurous acid is not in too large a proportion, the solution becomes yellow by dissolving by iodine; if more sulphurous acid is added, water is decomposed, and sulphuric acid, and hydroionic acid formed.

The *double compound* of potassium and iodine has no action on oxalic, acetic, sulphurous, or phosphorous acids, but when it is mixed with the triple compound it is instantly decomposed by them, and iodine set free.

The same double compound in its pure state is decomposed very slowly by muriatic acid; and to convert the greater portion into muriate of potassa (potassane) it is necessary that the acid should be very frequently distilled from it, and a part always remains unaltered; when mixtures of the triple and double compounds are exposed to the action of muriatic acid, potassane (muriate of potassa) is instantly formed; and, if the proper proportions are adopted, none of the double or triple compounds remain, and the results are potassane only and the oxychloric acid.

Mixtures of the triple or double compounds produce abundance of iodine when acted on by glacial hydrophosphoric acid gas; but the pure double compound affords only hydroionic acid gas, and this decomposition offers the best method which has yet occurred to me of procuring pure hydroionic acid. When the two substances are gently heated together, the hydroionic acid gas, which comes over in considerable quantities, forms a colourless solution when absorbed by water.

I have

I have endeavoured to ascertain the composition of the triple compound of potassium. Seven grains that had been dried at the temperature of boiling water heated to redness in a small crucible of platinum lost 2·2 grains. Seven grains heated to dull redness in a small tube of glass lost 1·7 grain; a minute portion of iodine condensed in the middle part of the tube, but no violet vapour was observed in the upper part of it, and there was a very slight appearance only of moisture, so that the loss of weight in this last experiment must be principally ascribed to the expulsion of oxygen.

On a comparison of the results of these two analyses, it appears very probable that this triple compound is composed of one proportion of iodine about 165, one of potassium 75, and six of oxygen 90; which is a composition exactly analogous to that of the hyper-oxymuriate of potassa. The quantities that I used in my experiments were too small to render these results more than approximations, yet the similarity of them to those presented by the hyper-oxymuriates, ought perhaps to render them more worthy of confidence.

I have attempted to obtain pure triple compounds from solutions of baryta and lime, and from magnesia diffused through water, by dissolving iodine in them by heat, and by evaporating the clear liquor until it began to deposit crystals. In this way I have procured substances which, when well washed in distilled water, afforded no iodine to nitric acid, which yielded chlorine and chlorionic acid when acted upon by muriatic acid, and which when distilled afforded much oxygen and some iodine, and left substances which appeared to be mixtures of the earths with compounds that afforded iodine to sulphuric acid, producing a smell of sulphurous acid gas, and which probably consisted of the metals of the earths united to iodine.

The triple compounds from lime and magnesia were soluble without affording iodine in sulphuric acid; but on evaporating the acid, at the time that the vessel of platinum in which the experiment was made became dry and almost red hot, the violet vapour was perceived. Even the triple compound from baryta did not afford iodine or oxygen by treatment with sulphuric acid, except under the same circumstances.

When I first discovered that the triple compounds dissolved in acids without effervescence, I thought it probable that the effect depended upon the formation of a compound of oxygen and iodine, similar to euchlorine, or the oxy-chloric acid, and which remained dissolved in the fluid; and on this idea I made a number of experiments with the hope of obtaining such a combination in a detached form.

I distilled the solution of the triple compound of potassium in sulphuric acid, but the only gaseous product I obtained was oxygen. Sulphuric acid and iodine condensed in the cool part of the apparatus, and the residuum was acid sulphate of potassa.

Conceiving that a compound of oxygen and iodine might nevertheless exist in the fluid, and be decomposable at a high temperature, ture, I attempted to obtain it by acting on the triple compound of barium by sulphuric acid, and by evaporating the fluid obtained at a gentle heat, and suffering it to cool at different periods of the process; but in this manner of operating I gained no better results.

The triple compound of barium is scarcely soluble in water. Water that had been boiled upon it gave only a slight cloudiness to sulphuric acid, which possibly might be owing to some double compound mixed with it: the fluid when evaporated nearly to dryness afforded fumes which had the characters of those of sulphuric acid, and by a red heat yielded iodine, and left sulphate of baryta.

When the solid triple compound of baryta was heated in very small quantities of diluted sulphuric acid, the fluid separated exhibited acid properties, and when gradually evaporated left a substance which congealed by cooling, and formed a solid of a yellow colour deliquescent in the air, strongly acid, and which reddened vegetable blues, and did not afterwards destroy them. When strongly heated, the substance afforded the same results as the substance procured from the fluid just mentioned.

The residual solid matter obtained by the action of sulphuric acid on the triple compound of barium was treated a second time with sulphuric acid, yet, when heated to redness, it yielded iodine in abundance.

I have repeated these experiments very often, because M. Gay Lussac has stated that an acid compound of oxygen and iodine may be procured by dropping sulphuric acid into a solution of the triple compound of barium; but the conclusions of this ingenious chemist seem to have been founded upon the want of effervescence in the process; and his experiments were made at a very early period of the investigation, and probably before this time he has found reason to alter his opinion.

It is probable that a binary compound of iodine and oxygen may be formed, but the facts presented by the action of acids on the triple compounds are not sufficient to prove its existence.

When small quantities of very diluted sulphuric acid are digested on the triple compounds of potassium and barium, the fluid obtained is always acid, and always precipitates muriate of baryta. I thought it possible that the compound of iodine and oxygen might possess this property; but on collecting the precipitate and examining it, it appeared to be a mixture of the triple compound and sulphate of baryta, and from all the facts it appears that in the action of acids on the triple compounds new combinations only are formed.*

I take

---

* When sulphuric acid is made to dissolve as much of the triple compound of potassium by heat as possible, the mixture congeals by cooling into a yellow transparent substance, extremely deli-

 quescent,

I take the liberty of proposing for the triple compounds the names of oxy-iodes, because, when decomposed by heat, they afford oxygen and iodine. Individually they may be named from their bases. Thus *oxy-potassame*, or oxy-iode of potassium, will signify the triple compound of potassium, oxygen, and iodine, and *oxy-barame*, or oxy-iode of barium, will denote the triple compound of barium.

*Some Observations on Hydroionic Acid, and on the Compounds procured by means of it.*—I have generally procured the hydroionic acid which I have used in my experiments by the process referred to in the last section, the action of hydrophosphoric acid on potassame, but I have sometimes employed the gas procured from moistened iodine by phosphorus.

The hydroionic acid gas is rapidly decomposed by being heated in contact with oxygen, and a solution of iodine and hydroionic gas in water is formed, and it is slowly decomposed by heat alone, affording a deep red-brown easily fusible substance, which seems to be a compound of hydroionic gas and iodine.

When condensed in water, it is instantly decomposed by solution of nitric acid and iodine precipitated.

The solution of hydroionic acid rapidly absorbs oxygen from the air, and becomes yellow, and at last deep tawny orange; and this absorption is assisted by light and heat, the hydrogen is attracted by the oxygen to form water, and the iodine formed is dissolved in the remaining acid.

The concentrated hydroionic acid will probably form a good eudiometrical substance; it does not render the vessels in which it is used cloudy like the hydrosulphurets by the deposition of solid matter, and it does not enlarge the volume of the residual air like some other substances.

The solution of the hydroionic acid is decomposed by being heated with the hyperoxymuriate of potassa, and iodine is produced.

Hydroionic acid gas, as I have mentioned in my last paper, is decomposed by all the metals I have exposed it to, except gold and platinum; and the same metals that decompose it in its gaseous state, likewise decompose it when it is in solution, requiring, how-

---

quescent, and very acid. On decomposing it by heat, *neutral* sulphate of potassa remains. Now, as oxygen and iodine are the only substances driven off by heat, it may be asserted that the acid property of the mixture depends upon these two principles; yet this conclusion does not follow according to sound chemical logic: iodine alone destroys the alkaline properties of potassa, and oxygen and iodine in combination with potassium form a difficultly soluble and almost tasteless substance. This substance, the triple compound, has only a weak attraction for sulphuric acid, and it might be expected that in combining with sulphuric acid it would not deprive it of its acid properties.

 ever, ever, in some cases, the assistance of heat. The fluid hydroionic acid tarnishes silver at common temperatures, and dissolves mercury slowly when boiled in contact with it.

It dissolves the alkaline and common earths, and forms with them compounds very analogous in their properties to the compounds they produce when acted on by muriatic acid.

I heated dry quick-lime in a small tube filled with hydroionic acid gas, a yellow fluid immediately formed, which was coloured by dissolving hydroionic gas and iodine, and a fusible compound soluble in water, and which had a bitter taste similar to muriate of lime, was produced.

I made the same compound by dissolving marble in the hydroionic acid; the compound when heated to redness became fluid, and when kept in fusion in contact with air emitted iodine, gradually lost its fusibility, and from being neutral became alkaline, so that at a high temperature iodine is partly expelled from calcium by oxygen. I proved this still more distinctly by fusing the compound in a close vessel, in which it was confined by mercury. There was no change. I admitted a little oxy-potassame, and caused it to give off oxygen by heating it: as soon as the calcareous compound was fused in contact with oxygen, it instantly emitted iodine, and lime was formed on the surface.

The compound formed from hydroionic acid and baryta is an acrid bitter substance, very similar in its taste to *barytane*, (fused muriate of baryta,) not decomposable when heated to whiteness unless oxygen is present, but, when it is heated in contact with oxygen, oxygen is absorbed, and a part of its iodine expelled.

Magnesia dissolved in hydroionic acid without effervescence, and the solution evaporated gave a solid substance, having a taste very similar to muriate of magnesia. Like that salt, it partly lost its acid by a red heat; but a portion remained not decomposable out of the contact of air, but which instantly afforded iodine when heated in contact with oxygen.

I dissolved glucina, ittria, and zircona, in the hydroionic acid; they formed neutral saline compounds. The compound of hydroionic acid and glucina was less soluble and more astringent in taste than the muriate of glucina, and was entirely decomposed when heated in the open air, affording hydroionic acid and iodine.

The compound formed from ittria was more soluble, and highly astringent; that formed from zircona astringent, with more of bitterness. Both these salts were decomposed when heated in the atmosphere, at a low red heat; a smell of hydroionic gas was perceived, iodine was produced, and the earths remained.

I mentioned, in a note dated Montpellier, Jan. 10, (containing a correction for my last communication to the Society,) that the alkaline property which I at first supposed to belong to the compounds of potassium and sodium with iodine, depended upon some undecomposed subcarbonate of potassa mixed with the hydrate of potassa I employed, as the subcarbonate of potassa is decomposed by iodine and carbonic acid set free, I had not thought it probable bable that the subcarbonate of potassa could interfere with this result. But I find that if the subcarbonate exist at all in the lixivium, a portion of it always remains undecomposed. I find likewise, that, when a solution of iodine in lixivium of potassa is rendered perfectly neutral, or even slightly acid by hydroionic acid, a strong red heat renders the solid substance obtained slightly alkaline, provided it be in contact with air. Whether the separation of iodine by oxygen, in this instance, depends upon some effect of the moisture contained in the atmosphere, or upon the continued action of fresh portions of oxygen on the same surface of the compound, it is not easy to say; but a similar effect I find is produced upon *potassile,* (fluate of potassa;) this substance gains the power of reddening paper tinged with turmeric, by being strongly heated in contact with the air.

The power of neutralizing acids does not belong to the true compound of iodine and potassium, but depends either upon the subcarbonate not decomposed, or upon the alkali formed during the ignition of the compound; the pure double compound seems to have no power of action on the acids it does not decompose; I fused it in contact with sulphurous acid gas confined by mercury in a glass tube, the salt gained a slight tint of yellow, but did not absorb its own volume of gas: after this, it slightly reddened litmus, so that the acid must have had little more than a mechanical adhesion to the salt.

When *potassame*, or iode of potassium, is fused with boracic acid, there is a perfect mixture of the two bodies. In my first researches on this mixture, I conceived that they entered into chemical union, and formed a violet-coloured glass, and that the acid property of the boracic acid was neutralized by the new compound; but I since find that the violet colour of the glass is owing to the development of iodine, and when the application of heat is long continued, much iodine is disengaged, and the colour of the glass changes to olive, and borate of potassa is formed. When the glass is dissolved in warm water, an olive-coloured power separates, soluble when boiled in the caustic alkalies, so that there is great reason to suppose that it is boron, and that the boracic acid is decomposed by the attraction of the potassium combined with the iodine for oxygen, assisted by the tendency of iodine to assume the elastic state.

I fused the neutral compound of iodine with silica; no change was effected when the experiment was made in close vessels, but when the mixture was exposed to air, and intensely heated, a little iodine was evolved, some potassa formed, and some silica dissolved by it.

*On other acid Compounds of Iodine.*—I have made several experiments on the combination of iodine and chlorine, obtained by admitting chlorine in excess to known quantities of iodine in vessels exhausted of air, and repeatedly heating the sublimate.

Operating in this way, I find that iodine absorbs less than one-third of its weight of chlorine.

The compound of iodine and chlorine is a very volatile substance, and, in consequence of its action upon mercury, I have not been able to determine the elastic force of its vapour. Hence the estimations of its composition from experiments on the quantity of chlorine absorbed in close vessels must necessarily be liable to error. In one experiment, in which I dissolved the sublimate, by admitting a small quantity of water into the retort, I found that eight grains of iodine had caused the disappearance of five and a quarter cubical inches of chlorine.

In another experiment, in which the sublimate was not dissolved by water, and in which the absorption was judged of by the admission of fresh quantities of the gas, twenty grains caused the disappearance of 9·6 cubical inches of chlorine, and the barometer being at 30·1, and the thermometer at 57° of Fahrenheit.

It seems probable, from these experiments, that the chlorionic acid consists of one proportion of iodine and one of chlorine.

The chlorionic acid formed by the sublimation of iodine in chlorine in great excess is of a bright yellow colour, when fused it becomes of a deep orange, and when rendered elastic it forms a deep orange-coloured gas. It is capable of combining with much iodine when they are heated together, its colour becomes in consequence deeper, and the chlorionic acid and the iodine rise together in the elastic state.

The solution of the chlorionic acid in water likewise dissolves large quantities of iodine, so that it is possible to obtain a fluid containing very different proportions of iodine and chlorine.

The pure solution of the chlorionic acid, when it is very diluted, loses its colour by being agitated for some time in contact with chlorine, and then, when poured into fixed alkaline lixivia or solutions of the alkaline earths, it causes the precipitation of substances having the characters of triple compounds of the oxyiodes. If it is coloured, or in its ordinary state, at the same time that the oxyiode is precipitated, much iodine appears, and it is impossible to render a *concentrated* solution colourless by agitation with chlorine, or to deprive it of its power of yielding iodine by acting on alkaline solutions. The chlorionic acid, when poured into a solution of muriate of baryta, causes a copious precipitate in it, which has all the characters of oxyiode of barium, and the liquor becomes very acid.

When the colourless solution of chlorionic acid is added to a strong solution of ammonia, a white powder is precipitated which detonates feebly by a gentle heat; and which, when decomposed in glass vessels, affords iodine and elastic matter which does not support combustion.

When highly-coloured chlorionic acid is employed, the powder that falls down is black, and detonates with much greater force, and by the slightest touch or motion, and it appears to be the same substance as that procured directly by the action of iodine on ammonia, and which I have shown to be a compound of azote and iodine. Whether the white powder is a similar substance containing taining a larger proportion of azote, or whether it is a compound of ammonia with oxygen and iodine, or with iodine and chlorine, I have not yet been able to determine.

It is soluble in excess of chlorionic acid, and in this way may be separated from the black powder; it affords a little moisture during its detonation, but it is not possible to say whether this is formed in the process, or whether it is water adhering to the compound, for the temperature of its decomposition is so low, that a proper degree of heat cannot be applied to render it dry.

When two bodies so similar in their characters and in the compounds they form, as iodine and chlorine, act upon substances at the same time, it is difficult to form a judgment of the different parts that they play in the new chemical arrangements produced. When I found that the chlorionic compound formed a strong acid by solution in water, I at first suspected that water was decomposed, and hydroionic acid and euchlorine formed; there was no effervescence in the process, and the proportions agree with the supposition; but I find that solution of euchlorine instantly decomposes hydroionic acid and precipitates iodine, which is afterwards redissolved by the chlorine set free; and nitric acid, which decomposes hydroionic acid, has no action on chlorionic acid.

It was possible likewise, that if water was decomposed, muriatic acid and a compound of iodine and oxygen might exist in the solution; I endeavoured to ascertain if this was the case by distilling the solution at different temperatures and collecting the products, but I obtained always the same fluid.

When coloured solution of chlorionic acid is boiled with hyperoxymuriate of potassa, it loses its colour, and chlorine is given off from it; but in this case it likewise gradually loses its acidity, and a substance which yields iodine by heat with much effervescence, and which is probably oxypotassame, is precipitated.

It appears to me most probable, that the acid property of the chlorionic compound depends upon the combination of the two bodies; and its action upon solutions of the alkalies and the earths may be easily explained, when it is considered that chlorine has a greater tendency than iodine to form double compounds with the metals, and that iodine has a greater tendency than chlorine to form triple compounds with oxygen and the metals.

When in the case of the action of the chlorionic compound on fixed alkaline lixivia the chlorine is not in great excess, much iodine is always set free, because as it is easy to perceive from the proportions in which they combine, there is not sufficient oxygen detached from the alkali by chlorine to form the triple compound; and if the estimation of the composition of oxypotassame given in the first section be accurate, supposing that none of the double compound of iodine is formed, a solution must contain five proportions of chlorine to one of iodine, to produce a triple compound without the precipitation of iodine. It is however most probable, that some double compound of iodine is always formed, as a solution tion must be extremely diluted indeed to contain five proportions of chlorine to one of iodine.

When the solution of chlorionic acid is poured into solution of muriate of baryta, water must be decomposed to furnish hydrogen to the muriatic acid, and oxygen to the triple compound, and in this case some double compound of iodine and barium must be formed and remain dissolved in the solution.

From the action of chlorionic acid on metallic solutions, I am inclined to believe that triple compounds of the common metals, oxygen and iodine may be formed by means of it. It occasions a copious precipitate without effervescence in the solution of sulphate of iron, and in the solution of nitro-muriate of lead, and tin, and of nitrate of copper, and from analogy it is probable that these precipitates consist of the metal, oxygen, and iodine.

I thought it probable from the rapid action of tin on iodine, that tin-foil would burn in the vapour of iodine; but on introducing it into the violet-coloured gas in a small retort made very hot, though the combination was instantaneous, yet no light was apparent.

I thought it possible that the acid properties of the compound of tin and iodine, which I have described in my last communication to the Society, might depend upon the decomposition of water and upon the formation of hydroionic acid. On this idea I distilled the solution of it in water, hoping, if hydroionic acid were formed, that I should obtain some in this process; but the fluid that came over was merely water coloured by a minute quantity of iodine, and the orange-coloured substance which remained when dissolved in water, exhibited the same acid properties as before, and combined with ammonia without affording any oxide.

The compound of iodine and iron when dissolved in water exhibited acid properties, but when the solution was distilled it yielded hydroiotic acid and deposited oxide of iron, and the entire solution acted on by ammonia, afforded an olive-coloured precipitate in great abundance.

*On the Action of some compound Gases on Iodine.*—I heated some iodine in a dry glass globe filled with sulphuretted hydrogen; there was a considerable absorption of gas, no sulphur was deposited, and a reddish-brown fluid was formed, which when thrown into water rendered it strongly acid, and deposited much sulphur; the water passed through a filter exhibited the properties of hydroionic acid.

It is evident from this experiment, that sulphur, iodine, and hydrogen, are capable of forming a triple compound.

I sublimed some iodine in dry olefiant gas; a little of a reddish-brown fluid was formed, but the greatest part of the iodine crystallized on the sides of the vessel in which the experiment was made. By repeating the process several times, more of the fluid was formed. It was volatile at a moderate heat, and gave a yellow tint to water, but did not render it acid, there was a very slight absorption of the gas.

Iodine sublimed in nitrous gas effected no change in it.

 When

When iodine was exposed to carbonic oxide it underwent no change, it was repeatedly sublimed in it in common day-light without undergoing the slightest alteration.

When the violet gas was formed by heating iodine in carbonic oxide, and the vessel exposed for some time to the direct solar rays, a small quantity of a limpid fluid which had an acrid taste formed in the vessel. An accident prevented me from ascertaining if any gas had been absorbed, but it seems probable from this result, that, like chlorine, iodine may be combined with carbonic oxide by the agency of light.

*On the Mode of detecting Iodine in Combinations, and on certain Properties of its Compound with Sodium.*—I have examined many of the marine productions of the Mediterranean, with the view of determining whether they contained iodine. The ashes of the fuci and ulvæ of this sea afford it in much smaller quantities than the *sel de varec*, and in a very few cases only have I been able to obtain evidences of its existence in them.

M. Berard was so good as to order a considerable quantity of the species of ulva, which abounds on the coast of Languedoc, to be burnt for me at his laboratory of Montpellier. The ashes consisted for the most part of common salt, but a small quantity of alkaline lixivium which was obtained from them, afforded a red fluid when acted upon by sulphuric acid, and a similar colour I found was produced, when a solution of subcarbonate of soda and common salt, containing a minute quantity of the compound of sodium and iodine was treated in the same manner by the acid.

One of the best tests of the presence of a very minute quantity of iodine in compounds, is their action upon silver. Water when it contains less than $\frac{1}{1000}$ part of its weight of the double or triple alkaline compounds of iodine tarnishes polished silver.

The effect produced by compounds of iodine may be distinguished from that produced by sulphurets or sulphuretted hydrogen by this circumstance, that solutions containing sulphurets or sulphuretted hydrogen, by being boiled with a little muriatic acid, no longer tarnish the metal, whereas solutions containing iodine still retain the power.

Amongst a number of sea weeds that were obligingly given me for examination, by Professor Viviani, of Genoa, the ashes of the following afforded slight indications of the presence of iodine:

| | |
|---|---|
| Fucus cartilaginous. | Fucus filamentosus. |
| ——— membranaceous. | Ulva pavonia. |
| ——— rubens. | ——— linza. |

In the ashes of the corallines and sponges, I could discover no evidences of the presence of the substance.

I have examined three specimens of alkali formed by the combustion of vegetables that grow on the sea shore, one from Sicily, one from Spain, and the third from the Roman states, but not one of them afforded any indications of the presence of iodine.

I evaporated a considerable quantity of sea water procured at Sestri of Levanto in Liguria, in a part of the bay remote from any source source of fresh water; but I could gain no unequivocal evidences of the presence of the compounds of iodine in it. The residual liquor after the common salt had been separated, did not act upon silver, nor colour sulphuric acid. The first crystals of salt which fell down when fused upon silver, appeared me to tarnish it more than the last; from which it appeared probable that they may have contained some triple compound of iodine, yet after being ignited, they did not colour sulphuric acid. When a large quantity of this water was electrized by a Voltaic apparatus, and the products separated at the positive pole collected in a small cup of gold, which was covered with cement, except in the interior and lower part forming the circuit, a yellow solution was obtained, which when it was exposed to the negative pole of a Voltaic apparatus, yielded a black powder fixed in the fire, and not unlike the compound formed by heating gold and iodine together; but the quantity was too minute to admit of analysis, aad a dark-coloured substance is likewise obtained by negatively electrifying oxymuriate of gold, and there can be no doubt but that this substance formed a principal part of the solution.*

If iodine exists in sea water, which there is every reason to believe must be the case, though in extremely minute quantities, it is probably in triple union with oxygen and sodium, and in this case it must separate with the first crystals of common salt.

Whether the superiority which the curers of fish and meat are in the habit of attributing to bay-salt over rock-salt, is at all connected with the presence of the compounds of iodine, is an inquiry perhaps worth making; and the results of Dr. Henry's elaborate investigation of the composition of different kinds of salts, do not preclude the possibility of the circumstance, though they certainly diminish the probability.

I rubbed pieces of beef that had been killed some days, with the double and triple compounds of sodium. They did not putrify; the one rubbed with the double compound became very tender and soft, and of a red-brown colour; that exposed to the triple compound hardened considerably, and became of a paler colour.

The triple compound has very little taste, and neither of the compounds seems to have any pernicious quality when received into the stomach. I fed a goldfinch with bread soaked in water, holdin solution the double compound for two days, and he drank water holding in solution the triple compound for three days, without apparently suffering any inconvenience.—*Phil. Trans.* 1814. *Part II.*

---

* Iodine, like chlorine, combines both with gold and platinum, when heated with them, or when they are exposed to them in its nascent state.

CRITICAL

